# Robust Post-Processing for Marine GNSS/INS Integration: An Adaptive RTS Smoothing Approach via Huber M-Estimation

**DOI:** 10.3390/s26134107

**Published:** 2026-06-28

**Authors:** Shengya Zhao, Pengfei Sun, Jichao Yang, Zhihui Yin

**Affiliations:** 1 College of Intelligent Systems Science and Engineering, Harbin Engineering University, Harbin 150001, China; zsy@ndsc.org.cn; 2 Deep Sea Technology Department, National Deep Sea Center, Qingdao 266037, China; 3 College of Geodesy and Geomatics, Shandong University of Science and Technology, Qingdao 266590, China; smallwisdom18@163.com; 4 College of Ocean Science and Engineering, Shandong University of Science and Technology, Qingdao 266590, China

**Keywords:** GNSS/INS integration, adaptive backward smoothing, error-state extended Kalman filter (ESKF), Huber M-estimation, weak GNSS signal

## Abstract

**Highlights:**

**What are the main findings?**

- An Adaptive Rauch-Tung-Striebel Smoother (ARTSS) based on Huber M-estimation is proposed to dynamically modulate smoothing gains using forward filtering innovations.
- The proposed method significantly suppresses measurement outliers and mitigates forward filtering divergence, improving 3D positioning accuracy by up to 48.05% (land vehicle) and 31.07% (shipborne) compared to the standard ESKF.

**What are the implications of the main findings?**

- The ARTSS algorithm provides a highly reliable and robust post-processing mathematical solution for precision positioning and attitude determination in complex marine and terrestrial environments with severe GNSS signal blockages.
- It effectively overcomes the inherent vulnerability of standard Minimum Mean Square Error (MMSE)-based smoothing algorithms to non-Gaussian, heavy-tailed noise.

**Abstract:**

GNSS/INS integrated navigation systems play a critical role in marine navigation, providing high-precision position and attitude information for moving platforms. However, in complex marine environments—such as occlusions caused by offshore engineering platforms—GNSS signal attenuation frequently leads to a rapid degradation of positioning accuracy. To address this issue in post-processing applications, this paper proposes an Adaptive Rauch-Tung-Striebel Smoother (ARTSS)-based GNSS/INS integrated navigation method. The proposed method first performs forward filtering using an Error-State Extended Kalman Filter (ESKF). Subsequently, an adaptive equivalent weight is dynamically constructed using the Huber M-estimation cost function based on the forward filtering innovations. This adaptive factor is utilized to dynamically modulate the smoothing gain in the backward pass, thereby effectively suppressing the interference of measurement outliers. To verify the effectiveness of the algorithm, comparative experiments are conducted using real-world land vehicle and shipborne kinematic datasets. Three methods are evaluated: the standard ESKF, the fixed-interval backward smoothing (RTSS), and the proposed ARTSS approach. The loosely coupled solutions from the Inertial Explorer (IE) software serve as the reference truth. Experimental results demonstrate that the proposed algorithm achieves significant improvements in positioning and attitude accuracy during GNSS signal outages. Specifically, compared with the conventional ESKF and RTSS methods, the 3D position accuracy of the shipborne experiment is improved by 31.07% and 6.97%, respectively, while that of the land vehicle experiment is increased by 48.05% and 8.67%. Therefore, the method presented in this paper effectively mitigates the accumulation of forward filtering errors and significantly enhances the accuracy, stability, and reliability of the integrated navigation system in complex environments.

## 1. Introduction

In recent years, autonomous marine vehicles, such as Unmanned Surface Vehicles (USVs) and Autonomous Surface Vehicles (ASVs), have played increasingly critical roles in complex maritime operations. The safe maneuvering and coordinated control of these platforms rely heavily on highly accurate and continuous pose estimation [1,2]. In open-sky environments, the integration of the Global Navigation Satellite System (GNSS) and the Inertial Navigation System (INS) provides an optimal solution [3,4]. While modern inertial sensor technologies have advanced significantly [5], a standalone INS still suffers from inherent bias accumulation. Fusing GNSS measurements with INS outputs via Kalman filtering effectively limits these accumulating errors, enabling high-precision positioning and attitude determination [6,7].

However, in GNSS-challenged marine environments—such as harbor areas severely obscured by offshore photovoltaic panels, dense ship masts, or beneath marine engineering platforms—GNSS signals are highly susceptible to severe blockage and multipath interference [8,9]. Consequently, GNSS receivers often output unreliable positioning solutions with non-Gaussian heavy-tailed noise, leading to rapid degradation of the integrated navigation accuracy [10,11]. When GNSS signals are completely unavailable, the navigation system depends entirely on the standalone INS. Although the INS can maintain short-term accuracy, its position and attitude errors diverge quickly over time without external corrections [12,13,14]. While some researchers have employed artificial intelligence or hybrid neural networks to compensate for INS drift during outages [15,16], mathematical smoothing remains the most reliable post-processing approach.

To maintain satisfactory positioning and attitude accuracy under poor GNSS signal conditions, the Rauch-Tung-Striebel Smoother (RTSS) is widely adopted for post-processing [17]. As a fixed-interval backward smoothing algorithm derived from the Kalman filter framework, the RTSS employs all measurement data within the entire time span to achieve minimum-variance state estimation [18,19]. Nevertheless, a critical limitation persists: standard Kalman filtering and RTSS are based on the Minimum Mean Square Error (MMSE) criterion, which performs poorly in the presence of measurement outliers and non-Gaussian noise [20,21]. If the forward filtering results are heavily degraded by GNSS measurement outliers in complex marine environments, directly applying such corrupted results for backward smoothing will inevitably impair the final trajectory accuracy [22].

To address the vulnerability to outliers, robust filtering strategies have been extensively researched. Approaches based on Mahalanobis distance have been proposed to detect and isolate observation outliers [23], while M-estimation theory, particularly the Huber cost function, has proven highly effective in mitigating GNSS outliers by dynamically down-weighting anomalous measurements during the filtering process [24]. Furthermore, robust Student’s t-based Kalman filters using variational Bayesian (VB) approaches have demonstrated strong performance in suppressing abnormal disturbances [25].

However, most efforts focus primarily on the forward filtering stage. Recently, robust backward smoothing has attracted increasing attention. For instance, Huang et al. proposed a robust RTSS framework based on the generalized Gaussian scale mixture using VB inference to jointly estimate the state and unknown heavy-tailed noise parameters [26]. He et al. developed a minimum error entropy (MEE)-based RTSS combined with Taylor series linearization to resist complex non-Gaussian noises [27]. Yu et al. utilized Markov chain Monte Carlo (MCMC) with a Gibbs sampler to sample the state vector and noise covariance matrices simultaneously [28]. In the specific context of GNSS/INS integration, segmented error-state RTS smoothing [29] and adaptive recursive RTSS based on recursive least squares (RLS) [30] have been proposed to mitigate GNSS signal outages.

Despite these significant advancements, existing robust smoothers share inherent limitations when applied to highly dynamic marine engineering environments. In terms of weight construction and acting stage, methods based on global iterations (e.g., VB, MCMC, or MEE) [26,27,28] tightly couple the outlier suppression mechanism with complex forward-backward recursive loops, or they directly inflate the state covariance matrix during the real-time forward filtering phase. This mechanism of outlier suppression leads to two major drawbacks: (1) modifying the forward covariance directly breaks the real-time filter’s consistency and optimal structural stability, and (2) the required global iterations drastically increase the computational burden, rendering them less practical for massive high-frequency marine data processing.

To overcome these limitations and clearly delineate the innovation boundary, this paper proposes an Adaptive RTS Smoother (ARTSS) based on Huber M-estimation. Unlike existing methods, the proposed ARTSS features a physically decoupled architecture: it constructs an element-wise equivalent weight matrix using the forward standardized innovations, but strictly confines the action of this weight to dynamically modulating the smoothing gain in the backward pass. This strategy physically isolates outlier suppression from the forward state prediction, effectively cutting off the backward propagation of corrupted states without sacrificing computational efficiency or forward covariance optimality.

This paper is organized as follows. Section 2 introduces the principles of loosely coupled GNSS/INS integrated navigation, the error-state extended Kalman filter, the standard fixed-interval backward smoothing, and the proposed adaptive backward smoothing strategy based on Huber M-estimation. Section 3 details the experimental equipment and presents a comprehensive comparative analysis of the three methods to verify the performance of the proposed algorithm. Finally, Section 4 concludes the paper.

## 2. Methodology

### 2.1. Loosely Coupled GNSS/INS Integration

A loosely coupled integration scheme is employed for the GNSS/INS system.

The system state vector δx(t) is defined as 21-dimensional, and the observation vector $z_{rGNSS}$ is 6-dimensional. Under this configuration, the continuous-time state equation of the integrated navigation system is formulated as [31]:

(1) $$\delta \dot{x}(t)=F(t)\delta x(t)+G(t)w(t)$$

where F(t) is the state transition matrix, G(t) is the system noise matrix, and w(t) denotes the process noise vector, which is generally assumed to be white noise.

The state vector δx(t) is defined as [31]:

(2) $$\delta x(t)={\left[\begin{array}{ccccccc} {\left(\delta r_{INS}^{n}\right)}^{T} & {\left(\delta v_{INS}^{n}\right)}^{T} & \phi^{T} & b_{g}^{T} & b_{a}^{T} & s_{g}^{T} & s_{a}^{T} \end{array}\right]}^{T}$$

Here, $\delta r_{INS}^{n}$, $\delta v_{INS}^{n}$, and ϕ represent the position, velocity, and attitude error vectors of the INS, respectively. The terms $b_{g}$ and $b_{a}$ denote the gyroscope and accelerometer bias vectors, while $s_{g}$ and $s_{a}$ are their respective scale factor error vectors.

The position measurement equation for the integrated system is given by [31]:

(3) $$z_{rGNSS}={\hat{D}}_{R}({\hat{r}}_{GNSS}^{n}-{\tilde{r}}_{GNSS}^{n})$$

where ${\hat{r}}_{GNSS}^{n}$ is the position vector derived from the INS, expressed as ${\hat{r}}_{GNSS}^{n}={\hat{r}}_{IMU}^{n}+{\hat{D}}_{R}^{-1}{\hat{C}}_{b}^{n}l_{GNSS}^{b}$, with $l_{GNSS}^{b}$ representing the lever-arm offset vector. The term ${\tilde{r}}_{GNSS}^{n}$ denotes the position vector of the GNSS antenna phase center obtained from the GNSS solution, defined as ${\tilde{r}}_{GNSS}^{n}=r_{GNSS}^{n}-{\hat{D}}_{R}^{-1}n_{rG}$, where $n_{rG}$ is the GNSS position error vector.

Similarly, the velocity measurement equation is defined as [31]:

(4) $$\begin{array}{l} z_{vGNSS}={\hat{v}}_{GNSS}^{n}-{\tilde{v}}_{GNSS}^{n}\approx \\ \delta v_{IMU}^{n}-(\omega_{in}^{n}\times )(C_{b}^{n}l_{GNSS}^{b}\times )\phi -C_{b}^{n}(l_{GNSS}^{b}\times \omega_{ib}^{n})\times \phi -C_{b}^{n}(l_{GNSS}^{b}\times )\delta \omega_{ib}^{b}+n_{vG} \end{array}$$

In this equation, ${\tilde{v}}_{GNSS}^{n}$ is the GNSS-derived velocity vector of the antenna phase center, denoted as ${\tilde{v}}_{GNSS}^{n}=v_{GNSS}^{n}-n_{vG}$, with $n_{vG}$ representing the GNSS velocity error vector. The INS-derived velocity vector ${\hat{v}}_{GNSS}^{n}$ is expanded as [32]:

(5) $$\begin{array}{ll} {\hat{v}}_{GNSS}^{n} & \approx v_{IMU}^{n}+\delta v_{IMU}^{n}-(\omega_{in}^{n}\times )C_{b}^{n}l_{GNSS}^{b}-(\omega_{in}^{n}\times )(C_{b}^{n}l_{GNSS}^{b}\times )\phi \\ & -C_{b}^{n}(l_{GNSS}^{b}\times )\omega_{ib}^{b}-C_{b}^{n}(l_{GNSS}^{b}\times \omega_{ib}^{b})\times \phi -C_{b}^{n}(l_{GNSS}^{b}\times )\delta \omega_{ib}^{b} \end{array}$$

The overall measurement model for the GNSS/INS integrated navigation system can be compactly written as [24]:

(6) $$z_{k}=H_{k}x_{k}+n_{k}$$

where $z_{k}$ is the combined position-velocity measurement vector, $n_{k}$ is the measurement noise vector, and $H_{k}$ is the design matrix, structured as [24]:

(7) $$H_{k}=\left[\begin{array}{ccccccc} I_{3} & 0_{3} & \left(C_{b}^{n}l_{GNSS}^{b}\times \right) & 0_{3} & 0_{3} & 0_{3} & 0_{3}\\ 0_{3} & I_{3} & H_{vG1} & -C_{b}^{n}(l_{GNSS}^{b}\times ) & 0_{3} & H_{vG2} & 0_{3} \end{array}\right]$$

with the sub-matrices defined as $H_{vG1}=-(\omega_{in}^{n}\times )(C_{b}^{n}l_{GNSS}^{b}\times )-[C_{b}^{n}(l_{GNSS}^{b}\times \omega_{ib}^{b})\times ]$ and $H_{vG2}=-C_{b}^{n}(l_{GNSS}^{b}\times )\mathrm{diag}(\omega_{ib}^{b})$.

### 2.2. Extended Kalman Filter Based on Error States

**Figure 1:**
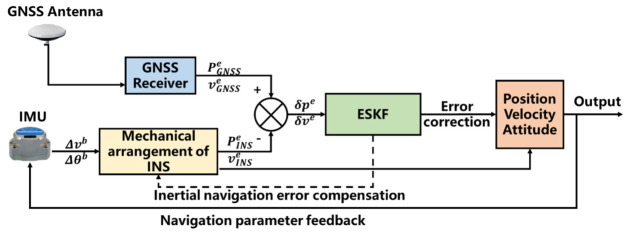
Architecture of the loosely coupled GNSS/INS integrated navigation system using the ESKF.

In loosely coupled GNSS/INS integration, the Error-State Kalman Filter (ESKF) is typically employed to compute the integrated navigation solution [33]. It is worth noting that while other advanced Bayesian filters, such as the Particle Filter (PF) and its variants like the Rao-Blackwellized Particle Filter (RBPF) [34], are theoretically superior at handling non-Gaussian noise, the loosely coupled GNSS/INS integration involves a high-dimensional state space (21 dimensions in this study). Applying PF in such a high-dimensional space inevitably causes severe particle degradation and unacceptable computational complexity for dynamic navigation. Therefore, the ESKF is selected as the baseline architecture due to its optimal engineering balance between applicability, computational efficiency, and standard accuracy. The corresponding algorithmic workflow is illustrated in Figure 1.

Derived from the continuous-time dynamics of the integrated navigation system, the discretized state and measurement models are formulated as [31]:

(8) $$\left\{\begin{array}{l} \delta x_{k}=\Phi_{k|k-1}\delta x_{k-1}+\omega_{k-1}\\ \delta z_{k}=H_{k}\delta x_{k}+n_{r,k} \end{array}\right.$$

where $\Phi_{k|k-1}=\exp(\int_{t_{k-1}}^{t_{k}}F(t)dt)$ is the state transition matrix, and $\omega_{k-1}=\int_{t_{k-1}}^{t_{k}}\Phi_{t_{k}|t}G(t)w(t)dt$ represents the equivalent discrete process noise vector. Here, G(t) denotes the continuous-time noise distribution matrix.

Given the established state and measurement models, the recursive execution of the ESKF [32] consists of two primary stages: the time update and the measurement update. During the time update (prediction) stage, the state vector and its corresponding error covariance matrix are propagated forward [32]:

(9) $${\hat{x}}_{k|k-1}=\Phi_{k|k-1}{\hat{x}}_{k-1}$$

(10) $$P_{k|k-1}=\Phi_{k|k-1}P_{k-1}\Phi_{k|k-1}^{T}+\Gamma_{k-1}Q_{k-1}\Gamma_{k-1}^{T}$$

Subsequently, upon the availability of a new GNSS observation, the measurement update (correction) stage is triggered. The Kalman gain is computed to optimally fuse the prediction with the new measurement, updating the state and covariance sequentially:

(11) $$K_{k}=P_{k|k-1}H_{k}^{T}{\left(H_{k}P_{k|k-1}H_{k}^{T}+R_{k}\right)}^{-1}$$

(12) $${\hat{x}}_{k}={\hat{x}}_{k|k-1}+K_{k}(z_{k}-H_{k}{\hat{x}}_{k|k-1})$$

(13) $$P_{k}=(I-K_{k}H_{k})P_{k|k-1}{\left(I-K_{k}H_{k}\right)}^{T}+K_{k}R_{k}K_{k}^{T}$$

### 2.3. Fixed-Interval Backward Smoothing Algorithm

**Figure 2:**
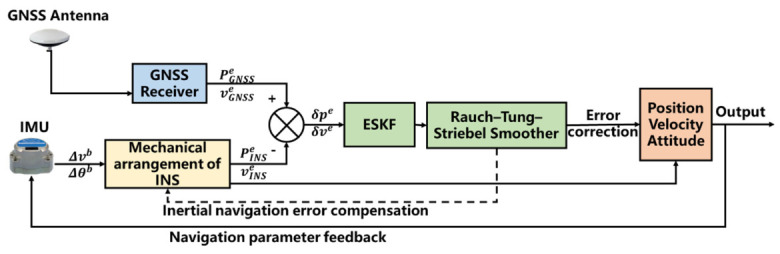
Flowchart of the GNSS/INS integrated navigation system incorporating the fixed-interval backward smoothing (RTSS) algorithm.

To refine the navigation trajectory, the state vector ${\hat{x}}_{f,k}$, state transition matrix $\Phi_{k+1|k}$, predicted covariance $P_{f,k+1|k}$, and updated covariance $P_{f,k}$ from the forward ESKF pass are preserved to facilitate the fixed-interval backward smoothing process. This study employs the Rauch-Tung-Striebel Smoother (RTSS) [35,36,37], an optimal smoothing algorithm that achieves minimum-variance state estimation by utilizing the full measurement context within a given time interval. The operational workflow of the RTSS integration is illustrated in Figure 2.

The RTSS algorithm executes in a backward temporal sequence, recursively updating the estimates from the end of the data interval to the beginning. First, the smoothing gain matrix $K_{s,k}$ at epoch k. is calculated:

(14) $$K_{s,k}=P_{f,k}\Phi_{k+1|k}^{T}P_{f,k+1|k}^{-1}$$

Subsequently, the smoothed state estimate ${\hat{x}}_{s,k}$ is derived by incorporating information from the smoothed state at the subsequent epoch k+1:

(15) $${\hat{x}}_{s,k}={\hat{x}}_{f,k}+K_{s,k}({\hat{x}}_{s,k+1}-{\hat{x}}_{f,k+1|k})$$

Finally, the smoothed error covariance matrix $P_{s,k}$ is updated to reflect the enhanced estimation accuracy:

(16) $$P_{s,k}=P_{f,k}+K_{s,k}(P_{s,k+1}-P_{f,k+1|k})K_{s,k}^{T}$$

In these expressions, the subscripts f and s denote the quantities associated with the forward filter and the results of the backward smoothing pass, respectively. Compared to real-time filtering, the RTSS provides a more stable and refined navigation solution by effectively utilizing the entire observation sequence.

### 2.4. Adaptive Backward Smoothing Algorithm

The standard Rauch-Tung-Striebel Smoother (RTSS) relies fundamentally on the Minimum Mean Square Error (MMSE) criterion, which operates intrinsically within the $L_{2}$ norm space. While mathematically optimal for purely Gaussian linear systems, the $L_{2}$ norm assigns quadratic penalties to measurement residuals. This characteristic renders the adaptive backward smoothing process highly vulnerable to non-Gaussian, heavy-tailed noise, which is frequently induced by GNSS signal blockages and multipath effects in complex marine environments.

To formulate a mathematically robust smoothing framework without discarding underlying kinematic information, this paper introduces an Adaptive Rauch-Tung-Striebel Smoother (ARTSS) strategy grounded in M-estimation theory.

#### 2.4.1. Standardized Innovation and Equivalent Weight

First, the multidimensional forward innovation vector $v_{k}$ and its theoretical covariance $S_{k}$ derived from the forward Error-State Extended Kalman Filter (ESKF) are defined as:

(17) $$v_{k}=z_{k}-H_{k}{\hat{x}}_{f,k|k-1}$$

(18) $$S_{k}=H_{k}P_{f,k|k-1}H_{k}^{T}+R_{k}$$

where $z_{k}$ is the measurement vector, $H_{k}$ is the design matrix, ${\hat{x}}_{f,k|k-1}$ is the forward predicted state, and $R_{k}$ is the measurement noise covariance.

Since position and velocity measurements have entirely different physical dimensions, directly using the absolute innovation $\left|v_{k}\right|$ to identify outliers is mathematically inadequate. Therefore, a standardized innovation vector ${\tilde{v}}_{k}$ is constructed. The i-th element of the standardized innovation is calculated as:

(19) $${\tilde{v}}_{k,i}=\frac{v_{k,i}}{\sqrt{S_{k,ii}}}\;$$

where $v_{k,i}$ and $S_{k,ii}$ are the i-th element of $v_{k}$ and the i-th diagonal element of $S_{k}$, respectively.

Applying the Iteratively Reweighted Least Squares (IRLS) principle of M-estimation, an element-wise diagonal equivalent weight matrix $B_{k}=\mathrm{diag}(\beta_{k,1},\beta_{k,2},\ldots ,\beta_{k,m})$ is constructed based on the Huber cost function. The i-th diagonal weight is:

(20) $$\beta_{k,i}=\left\{\begin{array}{ll} 1, & |{\tilde{v}}_{k,i}|\leq c\\ \frac{c}{\left|{\tilde{v}}_{k,i}\right|}, & |{\tilde{v}}_{k,i}|>c \end{array}\right.\;$$

where c is the robust threshold. Based on the asymptotic efficiency of standard normal distributions in Huber theory, c is theoretically set to 1.345 in this study to achieve 95% efficiency for clean Gaussian noise while strictly resisting heavy-tailed outliers, thereby avoiding the ambiguity of empirical manual tuning.

#### 2.4.2. Adaptive Smoothing Gain Modulation

In the standard RTSS, the smoothing gain and the smoothed state heavily rely on the forward updated state ${\hat{x}}_{f,k}$ and its covariance $P_{f,k}$. According to M-estimation theory, when measurement outliers occur, the theoretical measurement noise covariance should be equivalent to an inflated matrix ${\bar{R}}_{k}=R_{k}B_{k}^{-1}$.

If this inflated covariance is directly fed back into the real-time forward filter, it risks forward divergence, breaks covariance consistency, and increases the computational burden. To overcome this limitation, maintain the optimality of the real-time forward filter, and ensure that the backward smoother does not lock in the corrupted forward state, we propose an adaptive modulation mechanism based on backward-stage robust reconstruction.

Specifically, during the reverse-time smoothing pass, before computing the smoothing gain at epoch k, we utilize the standardized weight matrix $B_{k}$ to reconstruct a robustified equivalent forward Kalman gain ${\bar{K}}_{k}$:

(21) $${\bar{K}}_{k}=P_{f,k|k-1}H_{k}^{T}{\left(H_{k}P_{f,k|k-1}H_{k}^{T}+R_{k}B_{k}^{-1}\right)}^{-1}$$

Subsequently, a temporary outlier-free forward state ${\bar{x}}_{f,k}$ and its corresponding covariance ${\bar{P}}_{f,k}$ are reconstructed strictly within the smoothing stage, without altering the actual real-time filter outputs:

(22) $${\bar{x}}_{f,k}={\hat{x}}_{f,k|k-1}+{\bar{K}}_{k}(z_{k}-H_{k}{\hat{x}}_{f,k|k-1})$$

(23) $${\bar{P}}_{f,k}=(I-{\bar{K}}_{k}H_{k})P_{f,k|k-1}\;$$

Finally, these reconstructed robust components are utilized to modulate the smoothing gain $K_{s,k}$ and update the ultimate smoothed state vector ${\hat{x}}_{s,k}$ and its error covariance $P_{s,k}$:

(24) $$K_{s,k}={\bar{P}}_{f,k}\Phi_{k+1|k}^{T}P_{f,k+1|k}^{-1}$$

(25) $${\hat{x}}_{s,k}={\bar{x}}_{f,k}+K_{s,k}({\hat{x}}_{s,k+1}-{\hat{x}}_{f,k+1|k})\;$$

(26) $$P_{s,k}={\bar{P}}_{f,k}+K_{s,k}(P_{s,k+1}-P_{f,k+1|k})K_{s,k}^{T}$$

This mathematically rigorous treatment perfectly resolves the outlier propagation paradox inherent in conventional methods. When a severe GNSS outlier is detected ($\beta_{k,i}\to 0$), the equivalent noise covariance approaches infinity ($R_{k}B_{k}^{-1}\to \infty$). Consequently, the reconstructed gain ${\bar{K}}_{k}$ converges to 0. As a result, the reconstructed state naturally falls back to the pure kinematic prediction (${\bar{x}}_{f,k}\to {\hat{x}}_{f,k|k-1}$), entirely discarding the corrupted measurement $z_{k}$. The smoothed state ${\hat{x}}_{s,k}$ is then forced to rely exclusively on the robust kinematic prediction and the backward information propagated from the future, effectively isolating the errors and achieving high-precision continuous positioning under complex marine environments.

#### 2.4.3. Algorithm Implementation and Complexity

**Figure 3:**
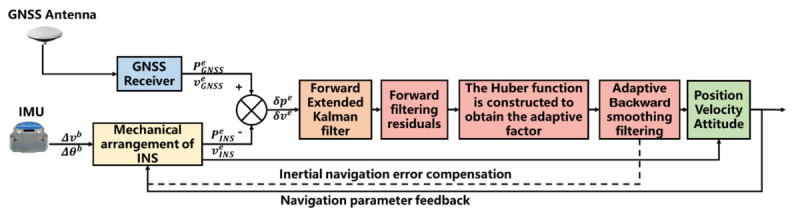
Flowchart of the proposed Adaptive Rauch-Tung-Striebel Smoother algorithm.

To clearly demonstrate the execution logic of the proposed algorithm, the overall system architecture is illustrated in Figure 3, and the detailed computational workflow of the ARTSS is summarized in Algorithm 1.
**Algorithm 1.** Computational workflow of the proposed ARTSSInput: Forward ESKF parameters: ${\hat{x}}_{f,k|k-1}$, $P_{f,k|k-1}$, $P_{f,k}$, $H_{k}$, $R_{k}$, $\Phi_{k+1|k}$, and raw measurement $z_{k}$ Output: Smoothed states ${\hat{x}}_{s,k}$ 1: For k=N−1 down to 0 do 2:  Compute standard innovation and its covariance using Equations (17) and (18) 3:  Calculate element-wise standardized innovation ${\tilde{v}}_{k,i}$ using Equation (19) 4:  Construct diagonal equivalent weight matrix $B_{k}$ using Equation (20) with c=1.345 5:  Reconstruct robust forward gain ${\bar{K}}_{k}$, state ${\bar{x}}_{f,k}$, and covariance ${\bar{P}}_{f,k}$ via Equations (21)–(23) 6:  Modulate smoothing gain $K_{s,k}$ via Equation (24) 7:  Update smoothed state ${\hat{x}}_{s,k}$ via Equation (25) 8:  Apply 360-degree cross-period correction specifically to the heading angle error component ($\phi_{U}$) within the state vector ${\hat{x}}_{s,k}$ to prevent angle wrap-around divergence:     If $\phi_{U}>180^{{}^{\circ}}$, then $\phi_{U}=\phi_{U}-360^{{}^{\circ}}$     If $\phi_{U}<-180^{{}^{\circ}}$, then $\phi_{U}=\phi_{U}+360^{{}^{\circ}}$ 9:  Update smoothed error covariance $P_{s,k}$ via Equation (26) 10: End For

Regarding the computational complexity, the proposed ARTSS is primarily dominated by the matrix inversions. The standard smoothing gain calculation requires the inversion of $P_{f,k+1|k}$, which takes $O(n^{3})$ operations (where n=21 denotes the state dimension). The proposed backward-stage reconstruction introduces the construction of the adaptive weight matrix $B_{k}$ and the inversion of the measurement innovation covariance $\left(H_{k}P_{f,k|k-1}H_{k}^{T}+R_{k}B_{k}^{-1}\right)$ during the gain reconstruction. Since $B_{k}$ only involves scalar standardizations and piecewise threshold judgments resulting in O(m) complexity, and the measurement matrix inversion takes $O(m^{3})$ (where m=6 denotes the measurement dimension), the added computational overhead is relatively minor. Given that m≪n, the overall asymptotic computational complexity of the proposed algorithm remains $O(n^{3})$. Consequently, the ARTSS provides strong robustness without introducing significant computational burdens compared to the standard RTSS, making it highly efficient for massive marine post-processing applications.

## 3. Experiments and Analysis

### 3.1. Land Vehicle Kinematic Test

**Figure 4:**
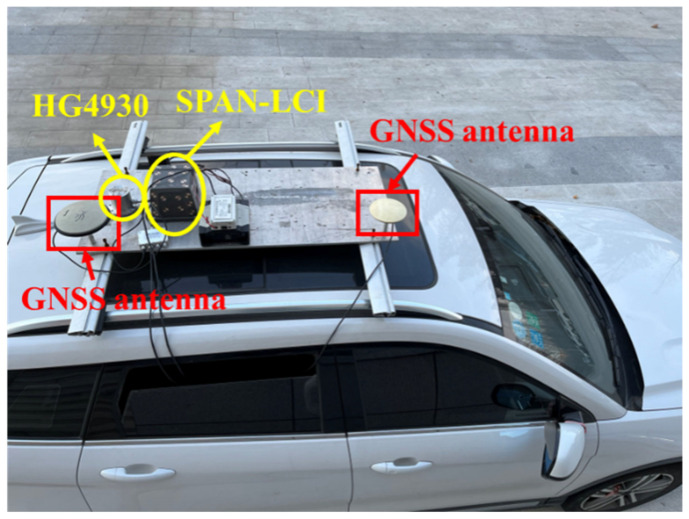
Test Vehicle and Experimental Equipment.

To verify the effectiveness of the adaptive backward smoothing algorithm, the test vehicle and sensors used in the experiments of this paper are illustrated in Figure 4.

The equipment parameters of the inertial sensors used in the self-developed POS system are listed in Table 1.

**Figure 5:**
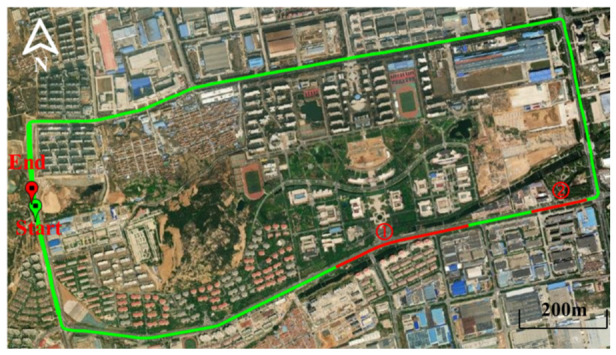
Rover Station and Motion Trajectory. Rover Station and Motion Trajectory. The green line represents the normal motion trajectory, while the red segments (marked as ① and ②) indicate specific regions where severe GNSS signal blockages or multipath effects occurred to evaluate the algorithm’s robustness.

The experimental trajectory started near the bus stop opposite the west gate of Shandong University of Science and Technology, circled around the university twice, passed by the south gate and the North Gate Food City, forming a rectangular trajectory, as shown in Figure 5.

### 3.2. Shipborne Kinematic Experiment

**Figure 6:**
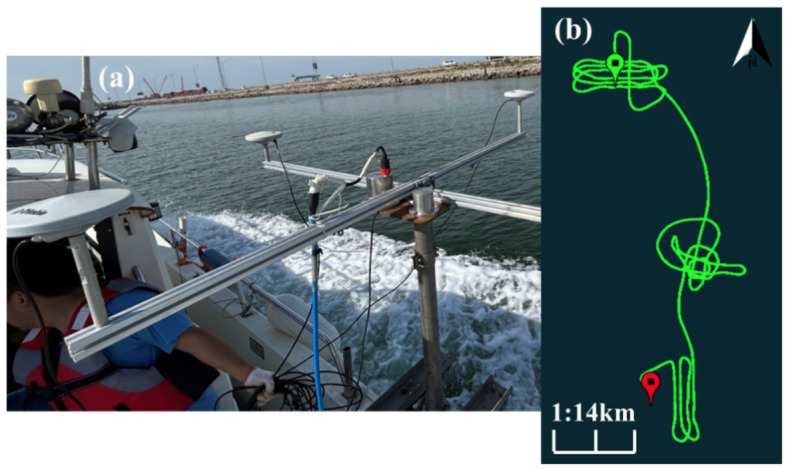
Shipborne kinematic experiment: (**a**) Experimental equipment using Honeywell HG4930; (**b**) Shipborne experimental motion trajectory.

To further verify the effectiveness of the algorithm in marine weak signal environments, shipborne data were adopted for experimental validation in this paper. The HG4930 manufactured by Honeywell (Phoenix, AZ, USA) was used as the experimental equipment, as shown in Figure 6a, and the experimental trajectory is illustrated in Figure 6b.

### 3.3. Experimental Results and Analysis

To systematically evaluate the effectiveness of the proposed algorithm, four comparative schemes are designed: Scheme 1 utilizes the high-precision post-processing solution generated by the commercial software Inertial Explorer (IE) 8.70 as the reference truth. The IE reference is computed using the Tightly Coupled (TC) Post-Processed Kinematic (PPK) dual-frequency carrier-phase algorithm combined with multi-pass optimal backward smoothing, achieving centimeter-level nominal positioning accuracy. Because our evaluated real-time algorithms (Scheme 2 to Scheme 4) strictly employ single-antenna Loosely Coupled (LC) pseudo-range and Doppler measurements, the reference truth and the evaluated algorithms are structurally and architecturally independent. Therefore, the evaluation bias is negligible, ensuring the high credibility of the comparative results.

#### 3.3.1. Land Vehicle Kinematic Results

**Figure 7:**
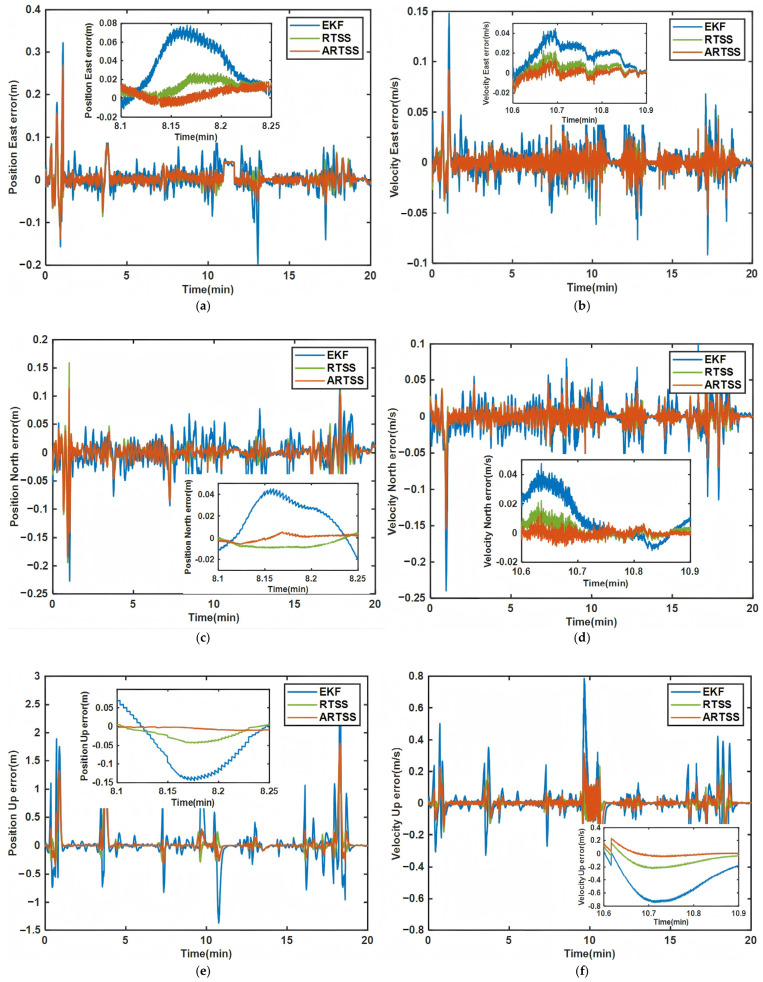
Position and velocity error results for land vehicle test: (**a**) Eastward position error; (**b**) Eastward velocity error; (**c**) Northward position error; (**d**) Northward velocity error; (**e**) Upward position error; (**f**) Upward velocity error.

To establish a baseline for the algorithm’s performance, land vehicle kinematic data are utilized for the experiments. The position and velocity errors are obtained by differencing the results of the ESKF, standard RTSS, and the proposed ARTSS with the high-precision reference truth, as illustrated in Figure 7.

Figure 7 illustrates the comparison of position and velocity errors in the East, North, and Up directions. Figure 7a,c,e present the position error comparisons, while Figure 7b,d,f display the corresponding velocity errors. Relatively large errors in the vertical (Up) direction primarily occur at the 2nd, 4th, and 18th minutes. This degradation is predominantly caused by weak GNSS signals resulting from signal obstruction by dense tree canopies and tall buildings. The standalone ESKF error is notably significant, reaching a maximum of 2.83 m. At the 18th minute, where the vertical position error peaks, the proposed ARTSS improves the accuracy by 22.22% and 60.00% compared to the standard RTSS and ESKF, respectively.

**Figure 8:**
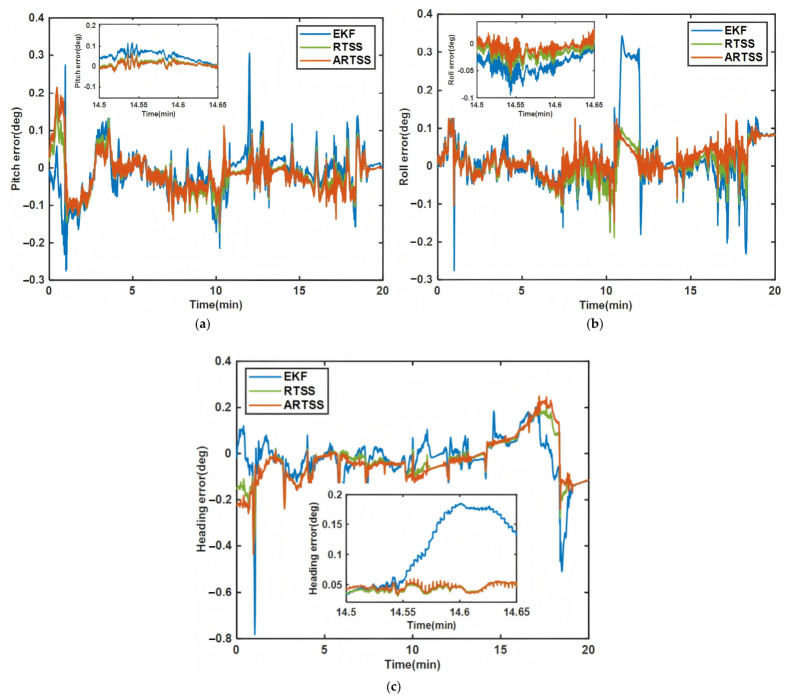
Comparison of attitude error results for land vehicle test: (**a**) Pitch error; (**b**) Roll error; (**c**) Heading error.

To further investigate the attitude error variations, the pitch, roll, and heading angle errors are depicted in Figure 8.

As shown in Figure 8, a noticeable roll angle error emerges near the 12th minute. The maximum roll angle errors for the ESKF, standard RTSS, and ARTSS are 0.34°, 0.12°, and 0.10°, respectively. Compared with the ESKF and RTSS, the accuracy of the ARTSS is improved by 70.58% and 16.67%, respectively. When the innovation vector from the forward filtering detects significant anomalies, the adaptive equivalent weight penalizes the outlier. Consequently, the backward smoother effectively dampens the corrupted state, thereby achieving high-precision positioning and attitude determination.

For a comprehensive comparison, the Root Mean Square (RMS) values of the position, velocity, and attitude errors for the three methods are summarized in Table 2.

Table 2 shows the RMS values of the position, velocity, and attitude errors. Compared with the standard RTSS and ESKF algorithms, the 3D position accuracy of the proposed ARTSS method is improved by 8.67% and 48.05%, respectively. The accuracy improvement relative to the standard RTSS is moderate, primarily because the forward filtering results remain reliable during most of the trajectory, allowing the standard smoothing to perform adequately. However, during severe GNSS signal outages caused by environmental occlusions, the adaptive factor effectively penalizes the degraded forward results. This adaptive intervention yields higher positioning accuracy and significantly enhances the stability and reliability of the navigation system.

#### 3.3.2. Shipborne Kinematic Results

**Figure 9:**
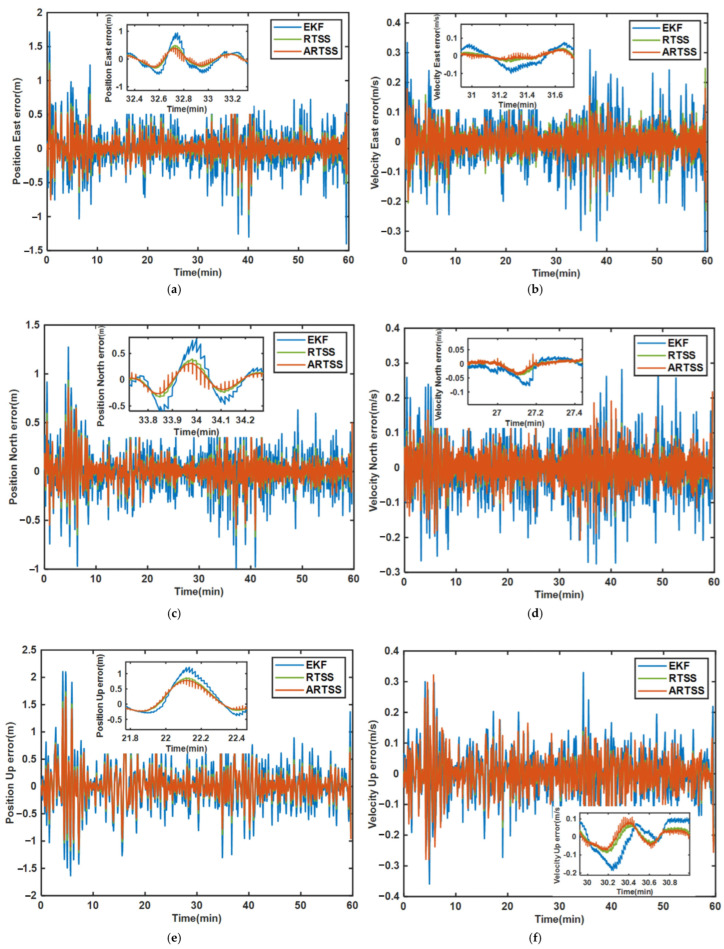
Position and velocity error results for shipborne test: (**a**) Eastward position error; (**b**) Eastward velocity error; (**c**) Northward position error; (**d**) Northward velocity error; (**e**) Upward position error; (**f**) Upward velocity error.

To evaluate the algorithm’s robustness in GNSS-challenged marine environments, a shipborne kinematic experiment was conducted. The position and velocity errors were obtained by differencing the results of the ESKF, standard RTSS, and the proposed ARTSS with the high-precision reference truth, as illustrated in Figure 9.

Figure 9 illustrates the comparison of position and velocity errors in the East, North, and Up directions. Figure 9a,c,e presents the position error comparisons, while Figure 9 b,d,f displays the corresponding velocity errors. Significant position and velocity errors in the vertical (Up) direction primarily emerge around the 6th minute. This degradation is largely attributed to the initial alignment instability of the equipment coupled with complex wind and wave dynamics. The standalone ESKF error is notably significant, reaching a maximum vertical error of 2.11 m. At the 8th minute, where the vertical position error peaks again, the ARTSS improves the accuracy by 21.82% and 32.23% compared to the standard RTSS and ESKF, respectively.

**Figure 10:**
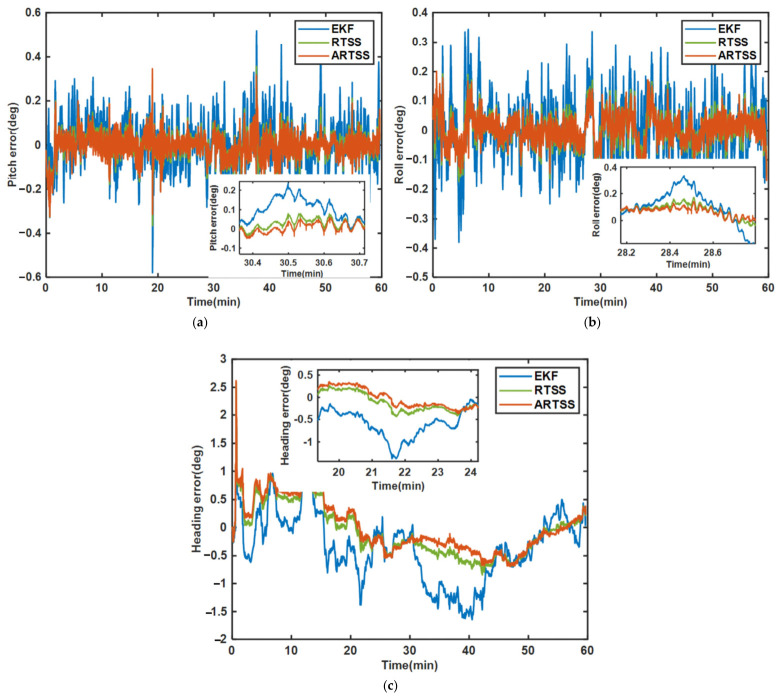
Comparison of attitude error results for shipborne test: (**a**) Pitch error; (**b**) Roll error; (**c**) Heading error.

To further investigate the attitude error variations, the pitch, roll, and heading angle errors are depicted in Figure 10.

As shown in Figure 10, the heading angle converges slowly under single-antenna conditions due to the intense influence of sea winds and waves, only beginning to stabilize after the 5th minute. At the 19th minute, the maximum pitch angle error for the ESKF reaches 0.58°, whereas the RTSS and ARTSS suppress it to 0.36° and 0.31°, respectively.

For a comprehensive comparison, the Root Mean Square (RMS) values of the position, velocity, and attitude errors for the three methods are summarized in Table 3.

Table 3 shows the RMS values of the position, velocity, and attitude errors. Compared to the ESKF and standard RTSS algorithms, the 3D position accuracy of the proposed ARTSS method is improved by 31.07% and 6.97%, respectively. The accuracy improvement relative to the standard RTSS is moderate, primarily because the forward filtering results remain reliable during most of the trajectory, allowing the standard smoothing to perform adequately. However, in this maritime scenario, the baseline distance between the rover and the base station extends to approximately 20 km, which, coupled with the complex wave-induced dynamics, severely degrades the GNSS differential positioning performance. By integrating the adaptive equivalent weight, the influence of the forward filtering divergence is dynamically penalized, yielding higher positioning accuracy and significantly enhancing the stability and reliability of the navigation system.

### 3.4. Discussion

Based on the comparative results of the land vehicle and shipborne kinematic tests, several potential benefits of the proposed ARTSS algorithm can be highlighted. First, by quantitatively integrating the standardized innovations into the backward pass to modulate the smoothing gain, the proposed algorithm establishes a closed-loop decoupled robust mechanism. This architecture effectively decouples the outlier suppression from the real-time forward filter, thereby resolving the divergence and covariance inconsistency issues inherent in traditional joint robust filtering. Second, from a practical perspective, the land vehicle experiment demonstrates that the 3D positioning accuracy of the ARTSS in urban canyons meets the sub-decimeter requirements. Similarly, the shipborne experiment confirms that heading angle errors are significantly suppressed under severe wave disturbances, which is of great value for autonomous vehicle navigation and automated ship berthing.

Furthermore, regarding the selection of the Huber tuning threshold c, a parameter sensitivity analysis was conducted during the preliminary tuning phase. We evaluated the 3D positioning performance across a range of threshold values (c=0.5, c=1.0, c=1.345, and c=2.0). The experimental results indicated that a smaller threshold (c=0.5) tends to be overly conservative, unnecessarily down-weighting normal innovations under high wave dynamics and compromising smoothing optimality. Conversely, a larger threshold (c=2.0) relaxes the robust boundary too much, failing to thoroughly suppress heavy-tailed measurement outliers induced by severe GNSS signal blockages. The selection of c=1.345 yields the minimum 3D root-mean-square errors (RMSE) in both vehicle and shipborne trials, striking an optimal balance between Gaussian efficiency and non-Gaussian robustness.

Despite these advantages, certain limitations of the current study should be acknowledged. On the one hand, although lever-arm compensation has been incorporated into the measurement equations, minor installation misalignments between the GNSS antenna phase center and the IMU center may still introduce unmodeled systemic biases under high-dynamic maneuvers. On the other hand, the experimental verification was conducted using a low-cost MEMS IMU (Honeywell HG4930). Under extreme sensor failure scenarios—such as sudden IMU bias jumps during prolonged GNSS blockages—the positioning error of the proposed method still exhibits short-term fluctuations. This indicates that highly non-Gaussian, non-stationary noises still impose extremely demanding requirements on the dynamic adjustment range of the robust boundaries.

To address these limitations, our future research directions will focus on several key aspects. We plan to integrate deep neural networks to assist in online noise characteristic identification, thereby enhancing the real-time modeling capability of non-stationary IMU noise during prolonged GNSS outages. Additionally, we will investigate more flexible probability distribution assumptions, such as skew-t or generalized Gaussian scale mixture distributions, to optimize the equivalent weight construction, further enhancing the algorithm’s robustness against highly asymmetric and impulsive disturbances.

## 4. Conclusions

To address the degradation of post-processing positioning accuracy caused by GNSS signal attenuation in complex environments (such as obstructions by tall buildings, tree canopies, or offshore engineering platforms), this paper proposes and verifies a reliable integrated navigation algorithm based on the Adaptive Rauch-Tung-Striebel Smoother (ARTSS). Building upon the forward predictions of the Error-State Extended Kalman Filter (ESKF), the proposed algorithm integrates Huber M-estimation to evaluate forward filtering innovations and construct an adaptive equivalent weight, thereby dynamically optimizing the smoothing gain during the backward pass.

Comparative experiments utilizing real-world land vehicle and shipborne kinematic datasets demonstrate that the ARTSS algorithm exhibits superior positioning and attitude determination performance. Specifically, in the land vehicle experiment characterized by dense signal occlusions, the 3D position accuracy of the proposed method is improved by 48.05% and 8.67% compared to the standard ESKF and RTSS, respectively. In the shipborne experiment, which was affected by complex wave-induced dynamics and long baselines, the 3D position accuracy is enhanced by 31.07% and 6.97%.

In summary, the adaptive backward smoothing method proposed in this paper effectively bolsters the system’s stability and reliability against measurement anomalies, significantly mitigates navigation error accumulation during GNSS signal outages, and provides a highly reliable post-processing solution for precision positioning and attitude determination in complex marine and terrestrial environments.

## Figures and Tables

**Table 1:** Parameters of HG4930 Equipment.

	Gyroscopes	Accelerometers
Bias	0.25°/h	0.025°/h
Random noise	0.04°/sqrt (h)	0.03 m/s/sqrt (h)

**Table 2:** RMS Values of Position, Velocity and Attitude Errors in the land vehicle test.

ERROR		ESKF	RTSS	ARTSS
Velocity (m/s)	Eastward	0.0151	0.0073	0.0064
	Northward	0.0214	0.0107	0.0085
	Upward	0.1099	0.0386	0.0294
Attitude (deg)	Pitch	0.0551	0.0478	0.0452
	Roll	0.0869	0.0064	0.0073
	Heading	0.0960	0.0955	0.0878
Position (m)	Eastward	0.0300	0.0208	0.0202
	Northward	0.0277	0.0177	0.0176
	Upward	0.3382	0.1919	0.1750
	3D Accuracy(m)	0.3407	0.1938	0.1770
	improved accuracy (%)	48.05%	8.67%	

**Table 3:** RMS Values of Position, Velocity and Attitude Errors in the shipborne test.

ERROR		ESKF	RTSS	ARTSS
Velocity (m/s)	Eastward	0.0757	0.0327	0.0362
	Northward	0.0746	0.0372	0.0342
	Upward	0.0732	0.0512	0.0496
Attitude (deg)	Pitch	0.1127	0.0506	0.0548
	Roll	0.1090	0.0513	0.0459
	Heading	0.6944	0.4623	0.4707
Position (m)	Eastward	0.2786	0.1905	0.1730
	Northward	0.2055	0.1487	0.1381
	Upward	0.4121	0.3172	0.2977
	3D Accuracy(m)	0.5382	0.3988	0.3710
	improved accuracy (%)	31.07%	6.97%	

## Data Availability

The raw data supporting the conclusions of this article will be made available by the authors on request.
